# Assessing the utility of advanced adoption models for AI-based financial services: insights into automated and hybrid robo-advisors

**DOI:** 10.3389/frai.2026.1854196

**Published:** 2026-07-14

**Authors:** Balraj Verma, Laxmi Remer, Divya Goswami, Somesh Kumar Sinha

**Affiliations:** 1 Chitkara Business School, Chitkara University, Punjab, India; 2 CBS International Business School, Cologne, Germany; 3 Rajagiri Business School, Kochi, India; 4 Rajagiri College of Social Science, Kochi, Kerala, India

**Keywords:** AI in financial services, fully-automated robo-advisor, hybrid robo-advisor, multigroup analysis, structural equation modeling, technology adoption models

## Abstract

**Introduction:**

Financial robo-advisors (FRAs), based on artificial intelligence (AI), are changing the ways people make investment decisions by providing advisory services that are based on algorithms and have different degrees of automation. But little evidence exists on the appropriateness of existing technology adoption theories to explain investor adoption of such systems. The present study investigates the explanatory and predictive power of three established and distinct adoption models for AI-enabled FRAs. It further develops a context-specific integrated adoption framework and examines whether adoption mechanisms differ between fully autonomous and hybrid robo-advisory services.

**Methods:**

For data collection, a quantitative research design was employed on 397 Indian investors through purposive sampling. The study uses explanatory and predictive assessment, integrated factor analysis, structural equation modeling, and multigroup analysis.

**Results:**

The findings reveal that the SVAM outperforms the TPB and UTAUT-II in terms of explanatory and predictive power and underscores the significance of value perceptions and self-efficacy in AI-enabled financial decision-making. The integrated framework identified attitude, hedonic automatism, perceived value, self-efficacy, performance expectancy, effort expectancy, and normative control perception as significant antecedents of behavioural intention, which in turn drives usage behaviour. The results also indicate that the impact of adoption drivers differs across types of robo-advisors, with self-efficacy playing a more significant role in fully autonomous settings, and attitude, effort, and performance-related evaluations gaining more importance in hybrid environments.

**Discussion:**

This study contributes to the literature on AI adoption by integrating competing theoretical perspectives into a context-specific framework and by establishing the type of FRA as an important boundary condition for investor behaviour.

## 1 Introduction

Financial decision making is undergoing significant transition as artificial intelligence (AI) reshapes how people engage with the investment management (Asemi et al., 2025). The rapid integration of AI into financial services, best notably via Financial Robo-advisors (FRAs), deliver data-driven algorithmic guidance with low to no human involvement, promising accessible, scalable, efficient, and cost-effective investment solutions (Nain and Rajan, 2023). By automating portfolio building and rebalancing, FRAs aim to democratize investing among a larger inexperienced or underserved investors (Barile et al., 2024; Goswami and Verma, 2024). However, the utilization of these platforms remains staggered, particularly in emerging markets as India where user-centric factors digital readiness, perceptions and contextual influences play a crucial role in driving the uptake (de Oliveira Santini et al., 2026). This led to a central theoretical concern emerged from this transformation: to understand how individuals form intentions to adopt AI-enabled financial decision systems in contexts where the cognitive control and accountability are dynamically shared between the human users and intelligent technologies.

The literature on technology adoption has expanded with a wide range of theoretical frameworks such as theory of reasoned action (TRA; Fishbein and Ajzen, 1975), theory of planned behaviour (TPB; Ajzen, 1991), unified theory of acceptance and use of technology (UTAUT-II; Venkatesh et al., 2012), technology acceptance model (TAM-III; Venkatesh and Bala, 2008), self-efficacy-based value adoption model (SVAM; Zhu et al., 2010) etc. during the last few decades. These frameworks provide valuable insights into the technology adoption across a variety of context and have consequently been employed to examine the FRAs adoption. However, despite the academic enquiry into FRAs has increased significantly, extent literature on FRAs remains theoretically scattered and methodologically siloed. Major focus has been on specific technological and behavioural constructs such as usefulness, performance and effort expectancy, facilitating conditions and social influence, commonly analyzed within the confines of specific adoption model. For instance, in context of digital financial services, Belanche et al. (2019) underline on the role of perceived behavioural control and attitude driving adoption, emphasizing on the importance of user-focused evaluations. Rahi et al. (2019) further utilize UTAUT to identify effort expectancy, performance expectancy, facilitating conditions and social influence driving adoption through attitude formation. Likewise, Yang et al. (2025) merge TAM with TTF to illustrate how Task-technology fit shapes user attitude, citing ease, usefulness and perceived risk as key antecedents. Nevertheless, extent literature on digital financial services context often depends on distinct theoretical lenses as discussed earlier, with little effort made to synthesize or compare determinants across models (Goswami and Verma, 2026; Verma et al., 2025; Sohn and Kwon, 2020). This poses a challenge since, technology adoption theories often lack consensus on a single best explanatory as well as predictive model, leading to inconsistent assessment about potential users’ adoption. Although these models have their extensive application across diverse digital environments such as mobile payments services (Hassaan and Yaseen, 2026), digital healthcare (Rouidi et al., 2022), wearable services (Han et al., 2026), e-commerce (Khan et al., 2026), the predictability strength of these models within the AI-driven financial services continues to be limited and underexplored. This widespread application supports their significance in Fintech, but also highlights the need for cross-model clarity in AI-enabled FRAs context.

The existing scholarship on the FRA adoption has largely relied on the single adopted frameworks to explain the user behaviour. Prior research has specifically focused on variables such as ease of use, perceived usefulness, performance expectancy, as factors of adoption intention (Goswami and Verma, 2026; FakhrHosseini et al., 2024; de Oliveira Santini et al., 2026). Although this approach provides vital insights, but leads to limited theoretical integration, resulting in fragmented understanding of the complex mechanisms that shape investors acceptance of AI-driven financial advisory services.

Furthermore, the literature typically fails to distinguish between fully-automated and hybrid FRAs, a difference that is becoming more and more important in the evolving landscape of AI-enabled financial services (Zhang et al., 2021). Hybrid FRAs are those where the human expert complements the algo-based recommendations, and Fully-automated FRAs, which primarily operates through machine intelligence with minimal human interventions (Zhang et al., 2021). The distinctions are specifically important within the AI-driven financial ecosystems where the varying levels of the human involvement might impact the perceptions of the investors (Akhtar et al., 2025).

Despite significant advances in FRAs adoption studies, notable gaps remain as follows: *first,* there is no cross-theoretical evaluation within a single context to assess model’s relative explanatory and predictability power. *Second*, different adoption models laid multiple factor that shapes user behavioural intention, yet there is a scarcity of studies that synthesize determinants from multiple models to build an integrated adoption framework (Sohn and Kwon, 2020). Almost every study confined their investigation within the boundaries of original frameworks, resulting in overlap, redundancy and exclusion of potentially important variables. Thus, this limits our understanding on the factors which consistently have a greater impact on behavioural intention. The current investigation fills this vacuum by assimilating key factors from the evaluated models and perform a multi-theoretical construct synthesis to ascertain the influential predictors of FRAs adoption intention. *Lastly,* the extent literature fundamentally treats FRAs as a homogenous platform, discounting the fact that users’ responses may vary substantially between hybrid and fully automated platforms. As fully automated FRAs rely solely on intelligent algorithms for decision-making and hybrid FRAs incorporates human intervention to provide a blended approach (Barile et al., 2024). This lack of modality level difference overlooks the differing perception of control, efforts, self-efficacy, usefulness, personalization and value perception between the two platform types. There are barely any empirical evidences examining to demonstrate how the impact of key adoption determinants varies depending on the level of embedded automation in the service platform.

*Based upon the aforementioned discussion, the first objective of this study is to assess and determine which adoption model best explains and predict investors’ behavioural intentions towards FRAs. The second objective is to identify the key determinants of behavioural intentions by integrating key determinants from various adoption models to propose an integrated adoption model in the current context. The third objective is to assess how the key determinants of investors’ behavioural intention vary in terms of their relative impact across FRAs types.*

The current study aims to offers meaningful contributions both theoretically and practically. Theoretically, it pioneers a robust multi-theoretical construct synthesis within a single study context, something rarely employed in AI adoption studies. It enriches Fintech adoption theory by incorporating critical factors such as perceived value and self-efficacy and by introducing a fresh modality-based perspective. Practically, the findings will offer great insights to aid FRAs developers and policy stakeholders on how to fine-tune data-driven advisory judgements to encourage user uptake, especially in digitally emerging nations like in India. Lastly, it extends the understanding about technology adoption by analyzing the differences in the adoption of hybrid and fully automated FRAs adoption mechanisms and deduces to more nuanced, modality-specific adoption processes within the wider FinTech ecosystem.

## 2 Theoretical underpinning

Technology adoption has been studied through a series of theoretical paradigms that aim to explain why people want to adopt or reject new technologies (Yadegari et al., 2024; Rahman et al., 2017). The transition from generic behavioural theories to technology-specific models implies a growing sophistication in understanding adoption as a multidimensional phenomenon, driven by cognitive appraisals, social influences, perceived control, value assessments and individual capabilities (Sohn and Kwon, 2020). All these theories agree on behavioural intention as the proximal predictor of technology use but differ substantially in the mechanisms they propose to explain this intention.

The earliest foundation perspective is The Theory of Reasoned Action (TRA), which proposes individual attitudes and subjective norms determine behavioural intention (Fishbein and Ajzen, 1975). Building on this logic is the Theory of Planned Behaviour (TPB) which adds perceived behavioural control, recognising that even in the presence of positive attitudes, people may not act when they feel constrained in their ability to perform the behaviour (Ajzen, 1991). These theories stressed the role of attitudinal and normative processes in the determination of behavioural intention.

With the percolation of information technologies, academic developed technology-specific frameworks to improve the explanatory precision. The Technology Acceptance Model (TAM) is based on two main factors affecting technology acceptance, namely perceived usefulness and perceived ease of use (Davis et al., 1989). Later versions, like TAM2 and TAM3, added social influence, experience, and facilitating mechanisms to explain adoption in the organisational and consumer settings (Venkatesh and Davis, 2000; Venkatesh and Bala, 2008). Likewise, the Unified Theory of Acceptance and Use of Technology (UTAUT) combined constructs from eight major adoption theories and showed high explanatory power from performance expectancy, effort expectancy, social influence and facilitating conditions (Venkatesh et al., 2003). Venkatesh et al. (2012) further extended the UTAUT framework by incorporating the mediating effects of hedonic motivation, price value and habit to provide a more comprehensive understanding of consumer technology adoption.

Parallel theoretical traditions point to additional determinants of adoption. Value-based Adoption Model (VAM) suggests that users adopt a technology when the perceived benefits outweigh the perceived sacrifices (Taso and Ho, 2026). On the other hand, Social Cognitive Theory emphasizes the importance of self-efficacy, an individual’s confidence in his/her ability to perform a task, as a key determinant of behavioural outcomes (Bandura, 1977; Koutroubas and Galanakis, 2022). VAM considers adoption as a decision to maximize value, whereas self-efficacy perspectives focus on users’ perceptions of their competence to interact with the technology (Taso and Ho, 2026).

The co-existence of these frameworks suggests that behavioural intention can be explained through different theoretically grounded lenses. Some models emphasise rational utility evaluations (e.g., TAM, UTAUT), others social and normative processes (TRA, TPB), while others highlight value perceptions (VAM) or individual capabilities (Social Cognitive Theory). Thus, different models can achieve similar predictive performance based on different antecedent structures. This leads to a fundamental theoretical question: which theoretical framework is the most appropriate to explain behavioural intention in a specific technological context?

This question is especially relevant in the context of AI-mediated decision-making services. Many well-established theories of adoption were developed when technology was primarily viewed as a tool to support human decision making. However, AI-enabled services are increasingly characterized by autonomy, adaptivity and decision-support capabilities that may alter the way that users evaluate usefulness, control, trust, value and competence (Yang et al., 2024). While the final outcome of interest is still behaviorual intention, the relative importance of different antecedents such as attitudinal evaluations, performance expectations, perceived value, self-efficacy beliefs, and facilitating conditions may change in AI contexts (Shao et al., 2025).

While individual adoption frameworks have been extensively used in previous studies, relatively fewer studies have systematically compared competing theories in the same empirical setting to test their relative explanatory and predictive powers (FakhrHosseini et al., 2024). This fragmentation hampers the development of cumulative knowledge because findings are scattered across different theoretical traditions. Consequently, the literature is yet to establish as to whether behavioural intention in AI-mediated contexts is best explained by attitudinal mechanisms, perceived control, performance-related expectations, value assessments, self-efficacy beliefs or integrated theoretical approaches (Sohn and Kwon, 2020).

Another important theoretical implication is the possibility of theoretical convergence. Although they are derived from different theoretical traditions, several constructs in different frameworks capture conceptually related dimensions of user evaluation. For example, both performance expectancy and perceived usefulness refer to beliefs about benefits of using technology, and both facilitating conditions and perceived behavioural control refer to the ability of users to perform the behaviour (Rahman et al., 2017). Therefore, identifying antecedents that consistently show explanatory relevance across frameworks may permit the development of a more parsimonious and comprehensive account of behavioural intention formation.

Thus, the present study is comparative in its theoretical approach. Instead of presuming the superiority of any particular framework, it compares well-known technology adoption models in a common empirical setting to evaluate their relative explanatory and predictive capabilities. The purpose of this approach is twofold. First, it identifies the strengths and limitations of competing theoretical perspectives for the case of AI-mediated robo-advisory services. Second, it provides a basis for constructing a unified explanatory model that integrates the most prominent antecedents from across frameworks, thus contributing to cumulative theory building in technology adoption research.

### 2.1 Rationale for model selection

The study evaluates the three most popular adoption frameworks, i.e., TPB, UTAUT-2, and SVAM. These models have been adapted for their relevance to AI-mediated technology systems such as FRAs (Sohn and Kwon, 2020). Among the multiple frameworks for technology adoption, TPB, UTAUT2 and SVAM were selected because they offer complementary but non-overlapping perspectives to understand the adoption of AI-mediated financial technology. TPB addresses attitudinal, social and control factors, UTAUT2 addresses specific cognitive assessments of technology (Wu and Liu, 2023), and SVAM addresses the value perception and self-efficacy of users, which are increasingly considered as crucial in AI-enabled decision settings (Cao et al., 2022). Furthermore, the integration of TAM and VAM leads to conceptual redundancy since UTAUT-2’s performance and effort expectancy constructs capture perceived usefulness and ease of use, and SVAM encompasses the core value-based mechanisms of VAM (Rahman et al., 2017; Sohn and Kwon, 2020). Thus, these three models provide a parsimonious yet comprehensive basis for comparison of explanatory and predictive capabilities in the context of AI-enabled financial robo-advisors.

## 3 Method

### 3.1 Research design

The study was conducted using a quantitative and cross-sectional design to assess the determinants that influence the behavioural intentions of investors towards financial robo-advisor. The research is divided into three analytical phases: First phase is represented by the assessment of adoption models to identify and compare their predictive power; second phase by the integrated factor analysis to develop an integrated adoption model in the current context of study; and third phase by the multi-group analysis to study the variability of determinants influencing users’ behavioural intentions across different types of FRAs. The model specific elements that influence behavioural intentions for the model under investigation are shown in Table 1.

**Table 1:** Theoretical models and their relationships.

Model	Determinants	Outcome	Notes
Theory of planned behaviour (TPB)	Attitude (AT), Subjective Norms (SN), Perceived Behavioural Control (PBC)	Behavioural Intention (BI) → Usage Behaviour (UB)	Focuses on intention formation.
Self-efficacy value adoption model (SVAM)	Perceived Value (PV), Self-efficacy (SE), Perceived Fee (PF)	Attitude (AT) → Behavioural Intention (BI)	Attitude mediates on behavioural intention.
The unified theory of acceptance and use of technology (UTAUT-2)	Performance Expectancy (PEX), Efforts Expectancy (EEX), Social Influence (SI), FCN (Facilitating Conditions), HMO (Hedonic Motivation), PVA (Price Value), Habit (HAB)	Behavioural Intention (BI) → Usage Behaviour (UB)	Habit and facilitating conditions influence Behavioural Intention and Usage Behaviour both.

### 3.2 Sampling design

Before the survey was conducted, the questionnaire was examined by academics from well-known universities and experts in artificial intelligence and Fintech. After incorporating the concerns raised, they approved the questionnaire. The study used purposive sampling technique which is a non-probability sampling technique that seeks to focus on a particular group related to the study objectives (Obilor, 2023). The choice of purposive sampling is in line with prior research on the adoption of FinTech and AI, which suggests that exposure, knowledge, and basic technology literacy are a pre-condition for conducting a meaningful assessment of algorithmic financial services (Goswami et al., 2025). The sample method is not based on the representativeness of the population, but on the theoretical importance of the issue and on the ability of the people to form an informed position on FRAs (Etikan et al., 2016). In order to address the issue of sample homogeneity, the study actively recruited participants from different FinTech events in different regions, resulting in diversity in age, education, investment experience and platform familiarity, thereby increasing internal validity and maintaining contextual diversity. Sample collection was much assisted by Fintech specific event organizers and associations. These events attract the interest of financial experts and technology-savvy individuals from a variety of demographics. At several events, wherever possible, the interaction session of short duration was organized with the organizers to explain the concept of AI based financial services. Additionally, the rationale and purpose of the study were explained during the interaction with all potential participants and they were assured that their comments would be used only for academic purposes and would be kept anonymous and confidential. Before survey administration, informed consent was gained and no personally identifying or sensitive information was collected. Such low-risk study involving minimum-risk and consenting participants was not constrained by getting a formal ethical approval from the institution. In addition, the study was conducted in compliance with the Declaration of Helsinki’s tenets and internationally recognized ethical guidelines for study involving human subjects. Of those identified, 422 agreed to participate in a self-administered survey conducted online and off-line. The survey lasted for several months. After removing incomplete and inconsistent responses, there were 397 valid responses left for the final analysis. Table 2 provides a brief overview of the demographics of the participants.

**Table 2:** Demographic profile.

Demographic characteristic	Categories	Count (percentage)
Gender	Male	288 (72.54)
	Female	109 (27.46)
Age group (in years)	18–30	92 (23.17)
	31–40	164 (41.31)
	41–50	93 (23.43)
	51–60	44 (11.08)
	Above 60	4 (1.00)
Qualification	Graduates	188 (47.35)
	Masters and above	209 (52.64)
Robo-advisor platform preference	Hybrid FRAs	201 (50.63)
	Fully-Automated FRAs	196 (49.37)

### 3.3 Construct operationalization

The survey starts with a brief overview to give respondents a sense of AI-driven investment services. The measuring items were adapted from TPB, UTAUT-2 and SVAM which were further modified to the context of this study. Participants were asked to rate their level of agreement or disagreement with each statement on a 5-point Likert scale, where 1 is strong disagreement and 5 is strong agreement (Kankaraš and Capecchi, 2025). A detailed literature review was conducted to generate all measuring items which resulted in the assessment of 53 items. The study compares the predictability powers of the models by assessing the direct effects of the independent variables on behavioural intention, thus the moderating variables, including age, gender, voluntariness and experience were left out. However, the determinants that had either direct or indirect associations with behavioural intention were kept as they were, in accordance with the guidelines set by the corresponding models.

## 4 Data analysis and results

### 4.1 Initial quality checks of data

Prior to analysis, preliminary assessments were conducted to confirm the dataset’s robustness and validity, addressing non-response and method biases (Podsakoff et al., 2024). To assess potential non-response bias, the study used the extrapolation method, grouping respondents into “early” and “late” groups based on response times. This approach assumes that late respondents are similar to non-respondents (Fakhreddin, 2023). The mean scores of the constructs for the two groups were compared using independent samples *t*-tests. The results did not reveal any statistically significant differences for any constructs (see Supplementary Appendix Table A1). The results indicate that non-response bias does not threaten the validity of the study. Moreover, two different approaches were used to test the common method bias (CMB). The single factor test of Harman was conducted by performing the exploratory factor analysis on all the measurement items. The results showed that the dominant factor did not account for most of the variance (less than 50%) (see Supplementary Appendix Table A2), indicating that common method bias is not a major concern (Podsakoff et al., 2003). Subsequently, the full collinearity approach was applied to CMB through the calculation of the variance inflation factor (VIF) values for all constructs. The VIF values were obtained by regressing each construct on all other constructs in the model based on the recommendations of (Kock and Dow, 2025), all values were below the threshold of 3.3 (see Supplementary Appendix Table A3). This suggests that common method bias is unlikely to influence the findings of this study.

### 4.2 Data analysis for comparing models

The study used Partial Least Squares (PLS) based Structural Equation Modeling (SEM) to compare the explanatory and predictive power of the Theory of Planned Behaviour (TPB), the Unified Theory of Acceptance and Use of Technology (UTAUT-2) and the Self-efficacy Value Based Adoption Model (SVAM) to evaluate the effectiveness of theoretical frameworks to explain investors’ behavioural intention (BI) towards AI-based financial advisors (Cheah et al., 2026).

#### 4.2.1 Assessment of reliability and validity

The measurement model was used to test the validity and reliability of the survey instrument. The high content validity of the measures used in this study was achieved by selecting and adapting items from the standardized scales reported in the literature to the context of this study. This adaptation demonstrates that these items have been tested and validated by many researchers before which again confirms their content validity. Three criteria were used to evaluate the convergent validity. First, all factor loadings should be above 0.7 (Chin, 1998). Second, the composite reliability (CR) should be above 0.7 (Chin, 1998). Third, the average variance extracted (AVE) should be above 0.5 (Fornell and Larcker, 1981). The results in Table 3 show good convergent validity with all factor loadings above 0.7, AVE values ranging from 0.650 to 0.840, and CR values ranging from 0.848 to 0.955. Reliability was also tested by Cronbach’s alpha. To verify internal consistency, Cronbach’s *α* coefficients for the extracted items were calculated. The values of all constructs were between 0.847 and 0.955 (> 0.7), indicating adequate reliability.

**Table 3:** Measurement model assessment (from established models).

Model	Construct	items	Estimate	α	CR	AVE
TPB/SVAM	Attitude	AT1	0.909			
		AT2	0.887	0.916	0.926	0.806
		AT3	0.897			
TPB	Perceived behavioural control	PBC1	0.889			
		PBC2	0.908	0.938	0.943	0.805
		PBC3	0.897			
		PBC4	0.894			
TPB/UTAUT2	Subjective norm	SN1	0.857			
		SN2	0.903	0.914	0.915	0.781
		SN3	0.891			
SVAM	Perceived fee	PF1	0.792			
		PF2	0.785	0.847	0.848	0.650
		PF3	0.841			
SVAM	Perceived value	PV1	0.916			
		PV2	0.887	0.928	0.930	0.816
		PV3	0.907			
SVAM	Self-efficacy	SE1	0.867			
		SE2	0.875	0.915	0.919	0.740
		SE3	0.886			
		SE4	0.810			
UTAUT-II	Effort expectancy	EEX1	0.822			
		EEX2	0.831	0.907	0.907	0.710
		EEX3	0.880			
		EEX4	0.837			
UTAUT-II	Facilitating condition	FCN1	0.872			
		FCN2	0.881	0.933	0.935	0.783
		FCN3	0.895			
		FCN4	0.892			
UTAUT-II	Habit	HAB1	0.927			
		HAB2	0.902	0.955	0.955	0.840
		HAB3	0.911			
		HAB4	0.926			
UTAUT-II	Hedonic motivation	HMO1	0.890			
		HMO2	0.888	0.943	0.945	0.811
		HMO3	0.907			
		HMO4	0.916			
UTAUT-II	Performance expectancy	PEX1	0.921			
		PEX2	0.901	0.933	0.935	0.827
		PEX3	0.906			
UTAUT-II	Price value	PVA1	0.869			
		PVA2	0.863	0.926	0.929	0.766
		PVA3	0.876			
		PVA4	0.893			
All Models	Behavioural intention	BI	0.923			
		BI2	0.906	0.953	0.953	0.836
		BI3	0.920			
		BI4	0.907			

#### 4.2.2 Assessment of predictive relevance and explanatory power

The models were evaluated in terms of Explanatory Power (Adjusted *R^2^*) and Predictive Relevance (Q^2^). The findings reveal varying degrees of effectiveness of the models in explaining the behavioural intention to adopt FRAs as indicated by the Adjusted *R^2^*. The Theory of Planned Behaviour (TPB) demonstrated moderate explanatory capability accounting for 58.2% of the variance. The Unified Theory of Acceptance and Use of Technology-2 (UTAUT-2) had a stronger explanatory power, explaining 70.8% of the variance. The self-efficacy-based value adoption model (SVAM) explained 73.4% of the variance (See Table 4), which was highest among the models under examination, thus confirming the importance of including self-efficacy and the value-based dimensions in the context of artificial intelligence. Besides the explanatory power, the predictive accuracy of the models was also assessed by the Q^2^ statistic. Values above zero show predictive relevance and the larger the value, the stronger the accuracy. All the individual models have reached “High” levels of predictive accuracy as shown in the same table. Importantly, SVAM (*Q^2^* = 0.641) showed higher predictive accuracy than UTAUT-2 (*Q^2^* = 0.635) and TPB (*Q^2^* = 0.576).

**Table 4:** Evaluation of explanatory and predictive capabilities of models.

Model	*R^2^* Adjusted	Interpretation (explanatory power)	*Q^2^*	Level	Interpretation (strength of prediction)
UTAUT-2	0.708	Moderate	0.635	High	Strong predictive accuracy
TPB	0.582	Moderate	0.576	High	Strong predictive accuracy
SVAM	0.734	Moderate	0.641	High	Strong predictive accuracy

### 4.3 Development of an integrated model to predict behavioural intention

To develop an integrated model for examining behavioural intention towards FRAs an integrated factor analysis was performed by grouping constructs based on conceptual similarities and statistical convergence. An exploratory factor analysis (EFA) was conducted on the 53 measurement items. The EFA was based on Principal Component Analysis with Varimax rotation and Kaiser normalization. Items with factor loadings or communalities less than the recommended cut-off point of 0.50 were excluded from further analysis. The retained factors were then grouped according to statistical convergence and conceptual similarity. Those constructs that converged on a common factor were aggregated into an integrated construct. Those constructs that had a clear empirical separation were kept separate and retained as independent determinants in the integrated model. Thus, Perceived Fee and Price Value converged into Perceived Value and were represented by a single Perceived Value construct, and Subjective Norm and Perceived Behavioural Control were merged into Normative Control Perception. The final integrated model was composed of Attitude (AT), Effort Expectancy (EEX), Hedonic Automatism (HAU), Normative Control Perception (NCP), Performance Expectancy (PEX), Perceived Value (PV), Self-Efficacy (SE), Behavioural Intention (BI) and Usage Behaviour (UB).

#### 4.3.1 Reliability and validity assessment of derived constructs

The evaluation of the integrated measurement model confirms high internal consistency and convergent validity for each of the extracted constructs. From the integrated model data (Table 5), the following results were obtained: all of the outer loadings were far above the threshold of 0.70 (ranging from 0.708 to 0.984). All constructs were reliable with Cronbach alpha values ranging from 0.917 to 0.981 which are above the recommended level of 0.70. Also, the composite reliability values were always good, from 0.934 to 0.986, which further confirms the reliability of the integrated scales. The convergent validity was verified as all the AVE values were greater than 0.50 ranging from 0.671 to 0.947. The study also revealed the absence of multicollinearity since all the VIF values presented in the table were less than the cut-off point of 3.3 (Kock and Dow, 2025).

**Table 5:** Measurement model assessment (integrated model).

Construct	Scale items	Outer loadings	Cronbach’s alpha	CR	AVE	VIF
Effort expectancy	EEX1	0.881	0.924	0.946	0.814	1.149
	EEX2	0.89				
	EEX3	0.939				
	EEX4	0.896				
Attitude	AT1	0.968	0.954	0.97	0.916	1.816
	AT2	0.946				
	AT3	0.956				
Hedonic automatism	HAU1	0.85	0.938	0.949	0.698	1.832
	HAU2	0.822				
	HAU3	0.834				
	HAU4	0.841				
	HAU5	0.823				
	HAU6	0.825				
	HAU7	0.84				
	HAU8	0.851				
Normative control perception	NCP1	0.88	0.917	0.934	0.671	1.302
	NCP2	0.89				
	NCP3	0.874				
	NCP4	0.883				
	NCP5	0.708				
	NCP6	0.746				
	NCP7	0.739				
PEX	PEX1	0.984	0.972	0.981	0.946	1.713
	PEX2	0.965				
	PEX4	0.97				
Perceived value	PV1	0.967	0.967	0.976	0.911	1.89
	PV2	0.943				
	PV3	0.96				
	PV4	0.947				
Self-efficacy	SE1	0.927	0.939	0.956	0.845	1.471
	SE2	0.936				
	SE3	0.946				
	SE4	0.866				
Behavioural intention	BI1	0.982	0.981	0.986	0.947	1.000
	BI2	0.966				
	BI3	0.979				
	BI4	0.966				
Uses behaviour	UB1	0.973	0.977	0.983	0.935	
	UB2	0.966				
	UB3	0.977				
	UB4	0.952				

Discriminant validity was tested by the Fornell-Larcker criterion and the Heterotrait-Monotrait (HTMT) ratio. The square root of the average variance extracted (AVE) of each construct (shown in the diagonal of the discriminant validity matrix, Table 6) is higher than its highest correlation with any other construct, satisfying the Fornell and Larcker discriminant validity criteria. Similarly, the HTMT ratios (Table 6) were lower than the conservative thresholds, i.e., all values lower than 0.85 as proposed by Henseler et al. (2016), confirming the statistical distinctiveness of the constructs.

**Table 6:** Validity and reliability assessment of the derived constructs.

	AT	BI	EEX	HAU	NCP	PEX	PV	SE	UB
Fornell-larcker criterion									
**AT**	0.957								
**BI**	0.73	0.973							
**EEX**	0.257	0.397	0.902						
**HAU**	0.542	0.731	0.27	0.836					
**NCP**	0.387	0.493	0.154	0.387	0.819				
**PEX**	0.46	0.682	0.262	0.54	0.377	0.973			
**PV**	0.565	0.729	0.256	0.553	0.402	0.546	0.954		
**SE**	0.477	0.613	0.307	0.431	0.253	0.423	0.418	0.919	
**UB**	0.443	0.655	0.215	0.468	0.323	0.388	0.494	0.358	0.967

	AT	BI	EEX	HAU	NCP	PEX	PV	SE
HTMT criteria								
BI	0.751							
EEX	0.27	0.415						
HAU	0.571	0.761	0.289					
NCP	0.409	0.518	0.159	0.418				
PEX	0.475	0.699	0.275	0.565	0.396			
PV	0.586	0.748	0.27	0.58	0.423	0.564		
SE	0.502	0.636	0.326	0.454	0.261	0.439	0.434	
UB	0.457	0.669	0.225	0.489	0.345	0.398	0.508	0.373

Source: Authors’ calculations. The bold values represent the square root of the Average Variance Extracted (AVE), while the off-diagonal values represent the inter-construct correlations among the respective variables. AT = Attitude; BI = Behavioural Intention; EEX = Effort Expectancy; HAU = Hedonic Automatism; NCP = Normative Control Perception; PV = Perceived Value; PEX = Performance Expectancy; SE = Self-Efficacy; UB = Usage Behaviour.

#### 4.3.2 Structural model assessment of integrated model

**Figure 1:**
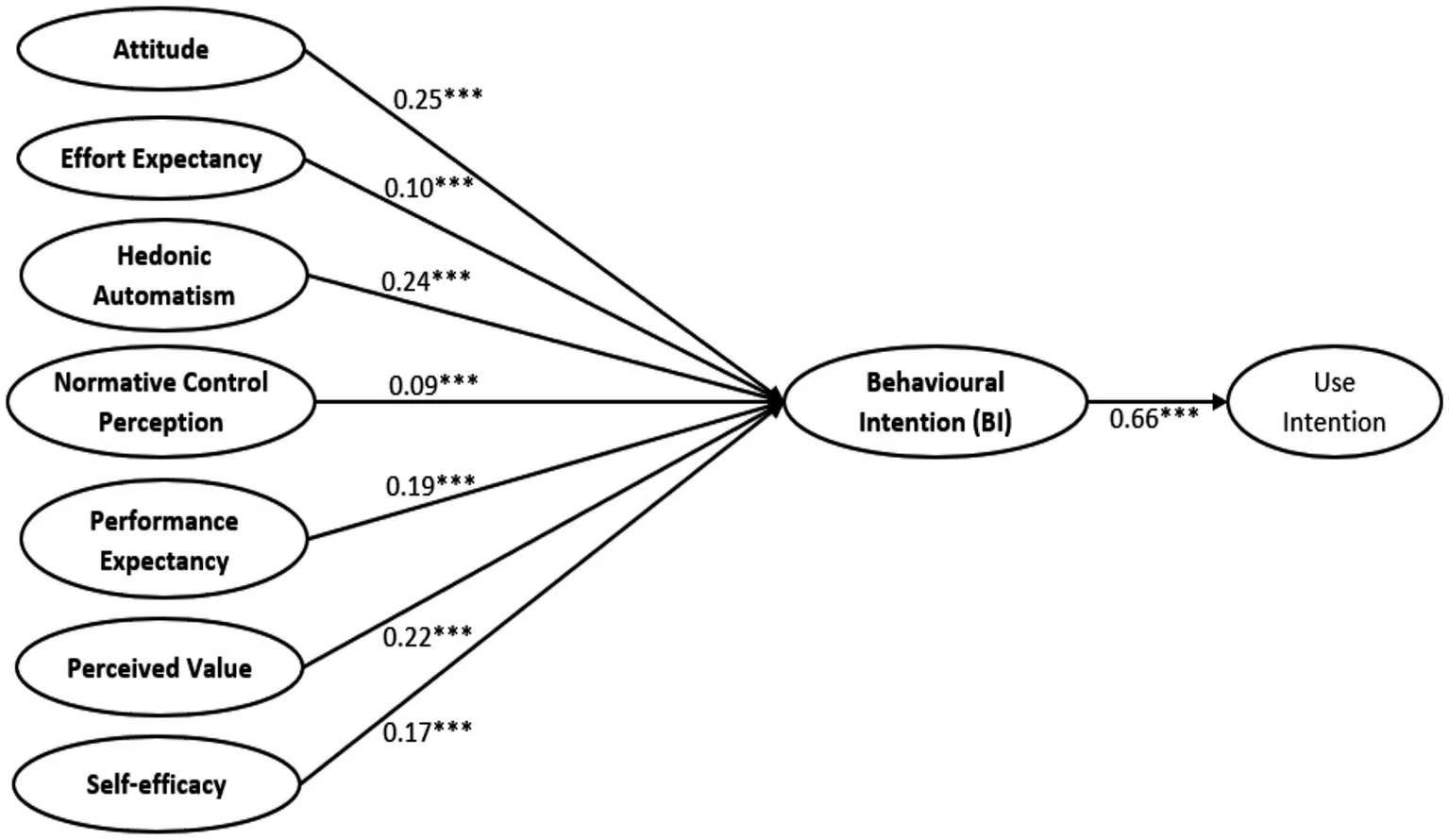
Unified adoption model for AI-enabled automated systems.

The structural model assessment shown in Figure 1 and Table 7 examines the direct relationships within the integrated model. The findings reveal that all determinants are positive and statistically significant. Attitude (AT, *β* = 0.249) emerges as the primary predictor of Behavioural Intention (BI), closely followed by Hedonic Automatism (HAU, *β* = 0.240), Perceived Value (PV, *β* = 0.224). Further, Performance Expectancy (PEX, *β* = 0.188) and self-efficacy (SE, *β* = 0.167) also exhibits meaningful positive impact. While factors such as Effort Expectancy (EEX, *β* = 0.097), and Normative Control Perception (NCP, *β* = 0.086) also exert significant influence on intention, their relative contributions within the model are relatively lower. Importantly, the relationship between Behavioural Intention and Usage Behaviour (UB) constitutes the most prominent path overall, with a β-value of 0.655 (*t*-stat. = 16.90), thereby confirming that an investor’s intention serves as a highly reliable predictor of the actual adoption of FRAs.

**Table 7:** Results of integrated structural model.

Paths	Beta coefficient	*t*-statistics	*p*-values	Result
Attitude → behavioural intention	0.249	6.496	0.001	Supported
Effort expectancy → behavioural intention	0.097	4.064	0.001	Supported
Hedonic automatism → behavioural intention	0.240	5.926	0.001	Supported
Normative control perception → behavioural intention	0.086	3.490	0.001	Supported
Performance expectancy → behavioural intention	0.188	5.172	0.001	Supported
Perceived value → behavioural intention	0.224	5.200	0.001	Supported
Self-efficacy → behavioural intention	0.167	5.942	0.001	Supported
Behavioural intention → usage behaviour	0.655	16.899	0.001	Supported

Besides the relevance of the individual relationships, the integrated model showed a high explanatory and predictive power, explaining 82.8% of the variance of behavioural intention (Adj-*R^2^* = 0.828) and showing a high predictive relevance (*Q^2^* = 0.821). The results indicate that the proposed integrated Model performs better than the individual adoption models in the current context of the study.

### 4.4 Importance-performance map analysis (IPMA)

**Figure 2:**
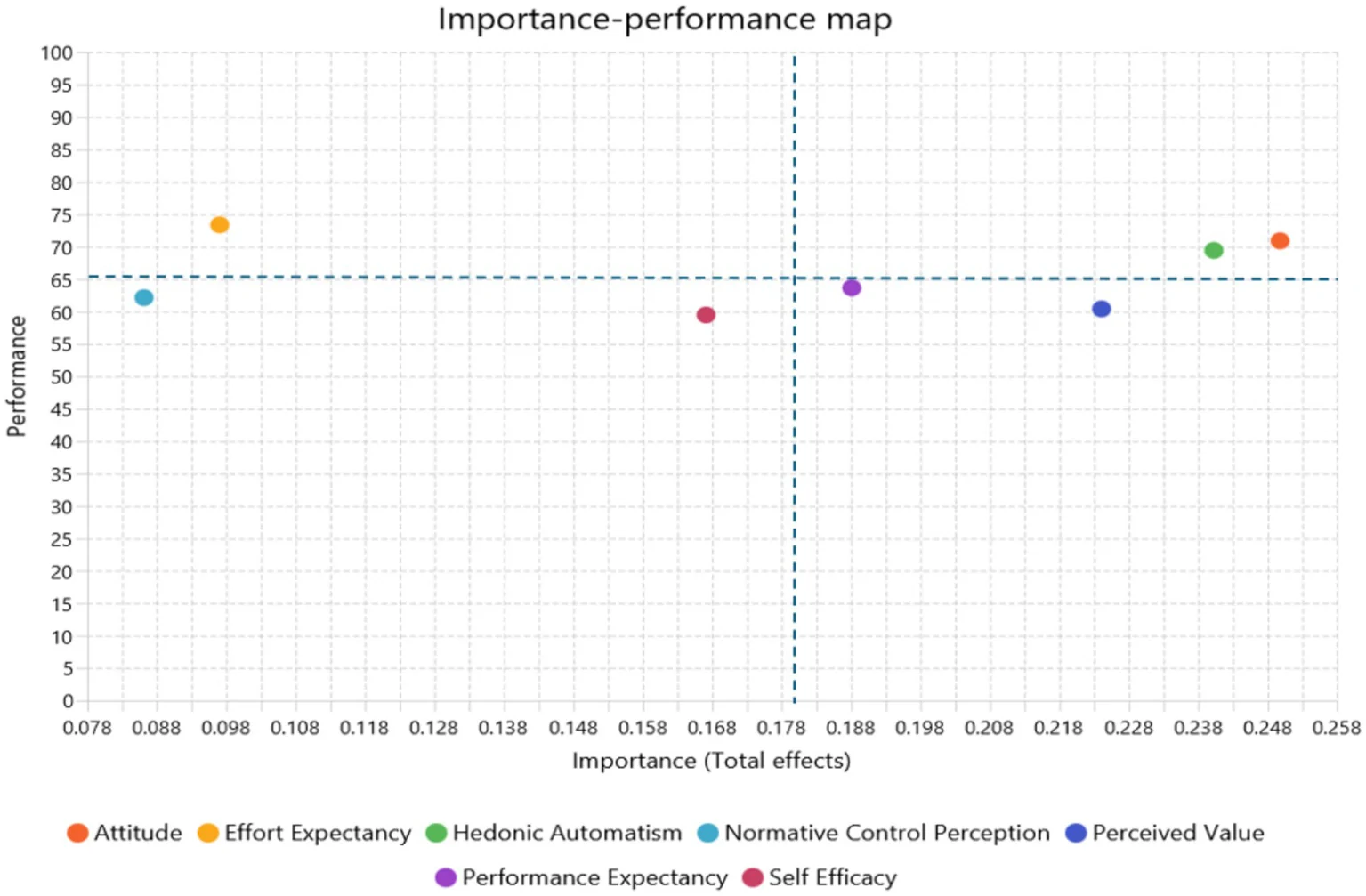
Important performance map analysis at the construct level.

The Importance-Performance Map Analysis (IPMA) classifies the primary determinants into four quadrants based on their importance and performance. The constructs are divided into four strategic quadrants according to the mean values computed (Importance = 0.179; Performance = 65.62) and shown in Figure 2.

Quadrant 1 (High Importance and High Performance): This quadrant includes: Attitude (AT) and Hedonic Automatism (HAU). These constructs are the major determinants of Behavioural Intention (BI) and are doing well for the time being. Attitude is users’ positive evaluation on the platform. Hedonic Automatism (HAU) is the joint effect of enjoyment and habitual use. It is suggested that organisations should retain and develop these qualities to sustain competitive advantage.

Quadrant 2 (High Importance and Low Performance): This quadrant contains Perceived Value (PV) and Performance Expectancy (PEX). These constructs are significantly affecting the Behavioural Intention (BI) but comparatively have lower performance levels. PV summarizes the cost benefit analysis. PEX is the expected performance of the functionalities of the platform. These are areas that need an immediate managerial focus and strategic resource allocation.

Quadrant 3 (Low Importance and Low Performance): This quadrant includes Normative Control Perception (PCB) and Self-Efficacy (SE). SE assesses how confident users feel about their ability to use the system. Normative Control Perception (PCB) is the combination of subjective norm and perceived behavioural control, its effect on Behavioural Intention (BI) is still relatively small and its performance is limited as well. As a result, it does not require immediate attention, but it should be watched for possible future significance.

Quadrant 4 (Low Importance and High Performance): This quadrant contains Effort Expectancy (EEX). EEX has a good performance but a quite low influence on Behavioural Intention (BI). This suggests that the system is likely to be fairly user-friendly already and that any additional investments in ease of use could be subject to diminishing returns. Resources might be more effectively allocated to other vital domains such as PV, PEX, and SE.

### 4.5 Multi-group analysis

To explore how the robo-advisor modality influences the relationship between fully automated-FRAs and Hybrid-FRAs, study conducted a multi-group analysis (MGA). The results showed a strong heterogeneity between the two advisory configurations as outlined in Table 8.

**Table 8:** Moderation effect across FRA subgroups.

Type of FRA	Fully automated-robo advisor			Hybrid-robo advisor			Multigroup analysis		
Paths	*β*-value	*t*-value	*p*-value	*β*-value	*t*-value	*p*-value	Difference	*p*-value	Result
Attitude → behavioural intention	0.106	2.241	0.025	0.282	4.408	0.000	−0.176	0.024	Supported
Effort expectancy → behavioural intention	0.013	0.876	0.381	0.125	3.467	0.001	−0.112	0.007	Supported
Hedonic automatism → behavioural intention	0.117	2.388	0.017	0.247	3.586	0.000	−0.130	0.120	Not Supported
Normative control perception → behavioural intention	0.026	1.382	0.167	0.100	2.226	0.026	−0.074	0.123	Not Supported
Performance expectancy → behavioural intention	0.080	2.413	0.016	0.236	3.234	0.001	−0.156	0.046	Supported
Perceived value → behavioural intention	0.096	2.297	0.022	0.237	2.979	0.003	−0.140	0.111	Not Supported
Self-efficacy → behavioural intention	0.663	5.057	0.000	−0.013	0.320	0.749	0.676	0.000	Supported
Behavioural intention → usage behaviour	0.630	11.647	0.000	0.684	12.226	0.000	−0.055	0.479	Not Supported

The effect of attitude was significantly higher among the antecedents of behavioural intention in the hybrid-FRAs (*β* = 0.282) than in the fully-automated FRAs (*β* = 0.106; Δ*β* = −0.176, *p* = 0.024). Similarly, effort expectancy has a stronger effect on behavioural intention for hybrid robo-advisor users (*β* = 0.125) compared to fully-automated FRA users (*β* = 0.013; Δ*β* = −0.112, *p* = 0.007). Furthermore, the effect of performance expectancy was significantly stronger in the hybrid setting (*β* = 0.236) as compared to the fully autonomous setting (*β* = 0.080; Δ*β* = −0.156, *p* = 0.046). These findings imply that investors engaging with hybrid robo-advisors weigh more heavily on positive appraisals of the system, perceived usability, and anticipated performance improvements in shaping their adoption intentions. One of the most important findings concerns self-efficacy. Self-efficacy had a very strong and significant influence on behavioural intention among users of fully autonomous robo-advisors (*β* = 0.663, *p* < 0.001), but not among users of hybrid robo-advisors (*β* = −0.013, *p* = 0.749). The difference between the two groups was highly significant (Δ*β* = 0.676, *p* < 0.001) and suggests that confidence in one’s ability to understand and use technology is significantly more important when investment decisions are delegated to an entirely automated advisory system. Rather, the presence of human intervention in hybrid robo-advisors seems to reduce the significance of individual technical proficiency.

However, the moderating effect of robo-advisor type was not supported for hedonic automatism, normative control perception, perceived value and the relationship between behavioural intention and usage behaviour. Although some of these constructs were significant in one or both groups, the differences in the path coefficients between groups were statistically insignificant (*p* > 0.05), implying that their effects remain relatively stable regardless of whether investors use a fully autonomous or hybrid robo-advisory service.

Overall, the results reveal that the adoption of hybrid robo-advisors is mainly influenced by utilitarian evaluations such as attitude, effort expectancy and performance expectancy, while the adoption of fully autonomous robo-advisors relies more on investors’ self-efficacy. These results highlight the importance of differentiating autonomous and hybrid advisory architectures in the study of investor acceptance of AI-enabled financial services.

### 4.6 Discussion

The first objective of the study was to compare the explanatory and predictive power of TPB, UTAUT2 and SVAM in explaining the adoption of AI-mediated financial robo-advisors by the investors. These results suggest that the psychological basis of adoption decisions is changing as technologies become more autonomous and algorithmic. TPB has demonstrated significant explanatory power in a number of technology contexts (Ajzen, 1991), but its comparatively weaker performance in the current study suggests that traditional attitudinal, normative and control-based explanations may be inadequate to account for investor decision-making in contexts where financial decisions are partially delegated to intelligent algorithms. Unlike traditional information systems, robo-advisers are not only applied to support the decision-making, but also increasingly participate in or even take over the decision-making processes on behalf of users (Krueckeberg, 2021; Nain and Rajan, 2023). That is, decision-making about adoption is less about how positively people feel about technology and more about whether they perceive enough value in algorithmic recommendations, and whether they have the confidence to effectively interact with such systems.

SVAM is another support to this argument by doing better. In contrast to TPB and even UTAUT2, SVAM explicitly integrates self-efficacy and value perceptions, which appear particularly relevant in AI-mediated financial contexts. Investment decisions involve uncertainty, risk and responsibility for the result. In such cases, investors are more inclined to assess not only the technological features of the system, but also whether the benefits that emerge from algorithmic advice are worth the cognitive, financial and psychological costs to utilize the system (Goswami and Verma, 2026). At the same time, as AI systems are often black-box decision agents, the users of such systems are expected to have a certain amount of faith in their ability to understand, control and correctly utilize the algorithmic outputs. This finding aligns with recent findings suggesting that self-efficacy and value-based reasoning are becoming increasingly important in AI-enabled technologies and intelligent service systems (Zhu et al., 2010; Cao et al., 2022; Sartono et al., 2024). The results thus contribute to the ongoing debate on the right theories in AI adoption research by indicating that higher algorithmic autonomy transforms the adoption decision from technology acceptance to technology delegation and at the same time calls for theories that can gather competence-based and value-oriented assessments as opposed to mere attitudinal evaluations.

The second objective was to develop and validate an integrated adoption framework. Our results suggest that cognitive, motivational, social and experiential mechanisms simultaneously influence the investor’s decision to use robo-advisors. Attitude remained a significant predictor of behavioural intention, suggesting that even with growing technological sophistication, investors are still making overall evaluative judgments about AI-mediated advisory services. Consistent with the literature on technology acceptance (TPB and related literature; Ajzen, 1991; Huang, 2023; Irimia-Diéguez et al., 2023), positive evaluations are likely to be indicative of beliefs that robo-advisors are a credible, reliable and modern way of managing investments.

Performance expectancy as another significant predictor can be understood with the fact that FRAs are judged on their perceived ability to enhance portfolio performance, investment recommendations and financial outcomes. User will embrace AI-enabled automated systems only if they see such systems more efficient than conventional human advisors, a result consistent with earlier literature (Zhang et al., 2021; Chung et al., 2023; Goswami and Verma., 2026). Further, the significance of effort expectancy indicates that technological advancement does not diminish the role of usability. Even tech-savvy investors still prefer platforms that minimizes complexity and support immersive interaction (de Oliveira Santini et al., 2026). Furthermore, perceived value is another significant predictor of behavioural intention. Investors seem to assess the benefits versus costs associated with any technology, which goes well with the value-based adoption model (Goswami and Verma, 2026; Cao et al., 2022; Zhu et al., 2010). In context to FRAs value is not merely an economic question rather a question of functional accessibility, efficiency and psychological reassurance. The result is supported by the fact that FRAs adoption is not decided by the novelty of the system, rather by the degree to which investors perceive notable advancements in their decision making.

The role of self-efficacy further lies in the uniqueness of AI-enabled FRAs. Financial decisions are taken in large uncertainty and reliance on algorithmic recommendations may lead to problems of loss of control and understanding. Thus, investors who are more confident in their ability to understand and interact with AI systems are more likely to adopt robo-advisory services. This finding is in line with findings from the AI product adoption and SVAM research, where self-efficacy remains a consistent crucial enabler of engaging with intelligent technologies (Sohn and Kwon, 2020; Cao et al., 2022; Taso and Ho, 2026). Further, the perception of normative control also significantly influenced behavioural intention, implying that the social influence and perceived control of behaviour become more closely linked in AI-enabled financial situations. In the future, as AI investing becomes more embraced by the financial markets, investors may be comforted by social acceptance and a sense that they have enough control over the use of technology. Similar observations have been reported in studies on FinTech and innovation adoption, where social validation increases confidence in emerging technologies (Ham et al., 2015; Irimia-Diéguez et al., 2023). Further, Hedonic Automatism, conceived as the integration of habit and hedonic motivation, extends traditional views of technology adoption by capturing the fact that repeated interaction with intelligent systems may result in conversion of deliberate use into automatic behavioural patterns. Conventional adoption theories mainly assume rational evaluation and conscious decision making (Davis et al., 1989; Ajzen, 1991). But smart financial platforms are getting better at providing frictionless, personalized, and always-on services that reduce the need for users to take action. Investors will be able to enjoy the results produced by the system in the long term, as well as the ease of investing using algorithms. Thus, convenience, automation and positive affect are psychologically associative. This finding supports new evidence on the joint effect of habit and hedonic motivation on digital technology use (Ren et al., 2026). More importantly, it also indicates that future research on the adoption of AI should go beyond purely utilitarian explanations and recognize that continued engagement with autonomous systems might increasingly be driven by automatic, experience-based behavioural mechanisms.

The results as the outcome of objective three reveal important theoretical boundary conditions for human-AI collaboration. Attitude, performance expectancy and effort expectancy were stronger in hybrid robo-advisory environments. This implies that, as long as human advisors are still involved in the decision process, investors are still assessing the technology according to the traditional technology assessment criteria (Nain and Rajan, 2023). Users seem to be more inclined to dwell on system usefulness, ease of use and overall evaluations, in line with traditional technology acceptance frameworks, as human expertise is still available as a safety net. In contrast, the importance of self-efficacy increased substantially in fully autonomous robo-advisory systems (Zhu et al., 2022). If decision-making authority is almost completely delegated to AI, investors can no longer seek explanations of recommendations or reassurance from human intermediaries. In this setting, confidence in one’s ability to comprehend, monitor and appropriately govern algorithmic decisions becomes crucial. Similar patterns have been found in research on the effects of different levels of technology autonomy, where higher levels of automation increased the importance of competence-related beliefs (Zhu et al., 2024). This finding adds to the literature on human-AI interaction by highlighting self-efficacy as an important psychological resource for users to maintain perceived control in highly autonomous settings.

Interestingly, the perceived value, normative control perception, hedonic automatism and the intention-usage relationship were invariant across both forms of advisory. This stability suggests that some mechanisms of adoption work independently of levels of autonomy (Glikson and Woolley 2020). Whether the advice is generated purely by AI or through human-AI collaboration, investors are still weighing general value, social legitimacy signals, and habitual engagement patterns. These results imply that while autonomy influences the relative importance of competence and performance evaluations, the overall motivational framework triggering the adoption of robo-advisors is surprisingly stable.

## 5 Implications

### 5.1 Theoretical implications

The study offers multiple significant theoretical contributions to the literature of technology adoption, human-AI interaction and FinTech. First, the study provides a comparative evaluation of TPB, UTAUT2, and the Self-Efficacy-Based Value Adoption Model (SVAM) for AI-mediated financial robo-advisors, which advances current discussions on the suitability of traditional technology adoption theories for increasingly autonomous digital environments. The superior predictive and explanatory power of SVAM suggests that when individuals entrust financially consequential decisions to automated AI-enabled systems, adoption intentions are less influenced by traditional technology-related perceptions, but more by the assessments of their own personal abilities and perceived value creation. This finding enriches the literature on technology adoption by indicating that AI-enabled financial decision-making technologies is a distinct adoption context in which users evaluate not only the functionality of the technology but also their own ability to interact with algorithmic recommendations and to derive meaningful benefits from them.

Second, the study contributes to the theoretical advancement by going beyond the boundaries of individual adoption models and designing an integrated adoption model primarily tailored to AI-enabled financial advisory systems. The identification of perceived value, attitude, effort expectancy, performance expectancy, self-efficacy, Hedonic Automatism and normative control perception as jointly influential determinants indicates that the complexity of AI-assisted financial decision-making cannot be adequately captured through single theoretical lens. Instead, user’s adoption behaviour in such environments reflects the interplay of cognitive evaluations, social influences and their own capabilities. Thus, the proposed framework provides a more context-specific understanding of technology adoption as well as establishes a robust foundation for future theorization in AI-mediated service ecosystems.

Furthermore, the study contributes to the contextualization of adoption theory. Robo-advisors are active participants in financial decision-making, unlike traditional information technologies, which are mostly passive. Therefore, users must trust, evaluate and sometimes delegate decisions to algorithmic agents. This transition from technology use to algorithmic dependence requires theoretical adaptations that incorporate delegation, autonomy, and perceived control. The integrated framework proposed in this study is a significant step towards such contextual adaptation and demonstrates the need for AI-specific extensions to the established technology acceptance models.

The results of the multigroup analysis further enriches the theoretical value by recognizing important boundary conditions of AI adoption processes. The stronger influence of self-efficacy in context of fully autonomous robo-advisory suggests that users are more likely to relinquish decision authority to algorithms when they are confident in their own ability to comprehend, evaluate, and use AI-based services. Conversely, the greater influence of attitude, effort expectancy and performance expectancy in hybrid advisory systems implies that users still assess these solutions through more traditional technology evaluation mechanisms where human involvement remains integrated into the service process. The study findings add to the emerging literature on human-AI collaboration by showing that the factors influencing adoption depend on the degree of algorithmic autonomy embedded within the system. Thus, the future AI adoption theories should explicitly incorporate automation level as a key contextual antecedent, rather than addressing AI-enable systems as a homogeneous category.

### 5.2 Practical implications

The findings have important implications for practitioners in the fast-changing robo-advisory ecosystem. Financial robo-advisor service providers should shift the emphasis from technical functionality to initiative building users’ confidence, perceived value and overall evaluation of AI-assisted investing. Structured onboarding processes should include guided demos, interactive tutorials and scenario-based simulations to help users understand how recommendations are produced and how investment decisions are endorsed. Such interventions reduce uncertainty and also increase engagement and long-term retention. With the advent of Hedonic Automatism, FinTech companies need to create experiences that are not only efficient but also enjoyable. UI should allow easy interaction with smart personalization, smooth portfolio monitoring, progress tracking and adaptive recommendations. Rather than making investing a transactional experience, firms should create engaging user journeys that will make investment management a rewarding, habit-forming experience. The findings are useful for traditional wealth management firms to strategically position hybrid robo-advisory solutions as complementary rather than substitutive mechanisms. Attitude, effort expectancy and performance expectancy had a more pronounced effect in hybrid settings, which indicates that investors still value the comfort of human expertise. Therefore, organizations must use the analytical power of the AI together with human advisory support to create collaborative service models that combine technological efficiency with interpersonal trust.

The results highlight the need for explainability, transparency and usability for AI designers and developers. Systems should be transparent about why they make recommendations, express the investment logic in plain language, and enable users to determine the level of detail they want to receive. These mechanisms can help to build confidence on the outputs of algorithm and help with informed decision making. These results also matter for investor education programs. Financial institutions, educational organizations and investor awareness programs should invest in developing AI literacy and digital investment competence. Workshops, simulation-based learning modules and practical training interventions can increase users’ self-efficacy and thus help to facilitate greater acceptance of autonomous financial technologies. The findings also underscore the importance of governance frameworks that promote the responsible deployment of AI to policymakers and regulators.” Regulators should pay attention to accountability mechanisms, transparency standards, safeguards for investor protection and ethical AI practices. With the right rules for algorithmic explainability and disclosure, we can create public confidence and sustainable growth in the robo-advisory industry.

Finally, the multigroup results point to different market segmentation strategies. Market fully autonomous robo-advisors as a theme of empowerment, competence-building, and autonomous wealth management. Focus on tools that give them confidence in operating independently with AI systems. In contrast, hybrid FRAs should be positioned on performance improvement, ease of use, and the combined benefits of human expertise and artificial intelligence. Such distinction positioning strategies can enhance market fit and adoption among different investor groups.

## 6 Conclusion

The study utilized a quantitative, cross-sectional design to examine the adoption of AI-enabled FRAs in India with data gathered from 397 investors using non-probability based purposive sampling. The study aimed at comparing the explanatory and predictive power of established technology adoption models, developing an integrated adoption model for FRAs and investigating relative impact of determinants in influencing behavioural intentions across robo-advisor types.

The study reveal that the self-efficacy-based value adoption model explains and predict investors’ adoption behaviour better than UTAUT2 and theory of planned behaviour. This means that the perceived value and the user competence are becoming more relevant in the AI driven financial environment. The study, therefore, built an integrated adoption model in the light of these insights, which combined cognitive, technological, and social and experiential determinants of behavioural intention. The results reports that attitude, hedonic automatism, perceived value, self-efficacy, performance expectancy, effort expectancy, and normative control perception, jointly affect investors’ intentions, which further lead to actual usage behaviour. The study also reveals that there are no consistent adoption mechanisms across the different FRAs configurations. The impact of key determinants differs between hybrid and fully-automated FRAs, indicating that investor assessments are contingent on the level of automation in the advisory process. This highlights the need for a more context-aware comprehension of AI adoption within financial services.

The study contributes to the technology adoption domain by developing a more appropriate explanatory as well as predictive model for AI-mediated financial systems and proposing an integrated model specific to FRAs. This can be useful for researchers, financial institutions and fintech providers who want to increase investor adoption and continued use of AI-enabled advisory platforms.

## 7 Limitations and directions for future research

Given its contributions, this study has several limitations that also provide opportunities for future research. First, the cross-sectional design of the research restricts the ability to make causal inferences and provides adoption perceptions at one point in time. As users’ interactions with AI-enabled financial systems are expected to evolve with experience, future research may use longitudinal designs to study how drivers of adoption evolve across pre-adoption, adoption and post-adoption stages. Moreover, the reliance on self-reported measures may lead to perceptual distortions and common method bias. Future studies could add behavioural, transactional or platform-generated usage data to survey-based evidence to improve robustness of findings. *Second*, the utilization of purposive sampling and targeting of existing and potential investors in India could restrict the generalizability of the results. The adoption of FRAs is embedded in broader cultural, technological and regulatory ecosystems that vary considerably across countries. Thus, future research should carry out cross-national comparisons with both developed and emerging economies to test the contextual robustness of the proposed model and uncover culture-specific adoption mechanisms. *Third*, the study uses three well-known frameworks of technology adoption, yet the theoretical lens is selective. Other perspectives like technology acceptance model, value adoption model, innovation resistance theory, diffusion of innovation etc. may offer additional insights into AI enabled financial decision making. Future research should examine how to integrate these perspectives to foster a broader comprehension of adoption and resistance toward AI-enabled advisory systems. *Finally*, the present study examined the Ai-mediated financial robo-advisory arrangements, future investigations may extend it to different AI-mediated contexts such as education, health care, autonomous mobility, customer service, recruitment, and generative AI platforms, could assist in establishing its external validity. Such extensions would add to a more nuanced and mature understanding of the ways in humans interact with increasingly autonomous AI systems.

## Data Availability

The original contributions presented in the study are included in the article/Supplementary material, further inquiries can be directed to the corresponding author.
