# Correction: Assessing the utility of advanced adoption models for AI-based financial services: insights into automated and hybrid robo-advisors

**DOI:** 10.3389/frai.2026.1948766

**Published:** 2026-08-11

**Authors:** Balraj Verma, Laxmi Remer, Divya Goswami, Somesh Kumar Sinha

**Affiliations:** 1 Chitkara Business School, Chitkara University, Punjab, India; 2 CBS International Business School, Cologne, Germany; 3 Rajagiri Business School, Kochi, India; 4 Rajagiri College of Social Science, Kochi, Kerala, India

**Keywords:** AI in financial services, fully-automated robo-advisor, hybrid robo-advisor, multigroup analysis, structural equation modeling, technology adoption models

Affiliation Chitkara Business School, Chitkara University, Punjab, India was erroneously given as Chitkara Business School, Chitkara University Chitkara Business School, Rajpura, India.

Affiliation Rajagiri College of Social Science, Kochi, Kerala, India was erroneously omitted for author Somesh Kumar Sinha.

The original version of this article has been updated.

