# Supplementary material for: Assessing the utility of advanced adoption models for AI-based financial services: insights into automated and hybrid robo-advisors
Source: Front Artif Intell. 2026 Jul 14;9:1854196. doi: 10.3389/frai.2026.1854196 (PMC13407770; doi:10.3389/frai.2026.1854196)
Supplement: Supplementary file 1 [file Table_1.docx]

**Appendix Table A1:** Non-Response Bias

| **Constructs** |  | **N** | **Mean** | **Std. Deviation** | **S.E. Mean** | **t** | **p-Value** |
| --- | --- | --- | --- | --- | --- | --- | --- |
| Perceived Value | EPV - LPV | 50 | -.05500 | .98236 | .13893 | -.396 | .694 |
| Perceived Fee | EPF - LPF | 50 | 0.13121 | 0.58154 | 0.10211 | 1.60522 | 0.11487 |
| Performance Expectancy | EPEX - LPEX | 50 | -0.1684 | 0.40425 | 0.07704 | -.899 | 0.39753 |
| Effort Expectancy | EEEX - LEEX | 50 | 0.01861 | 0.72246 | 0.12204 | 0.17419 | 0.86244 |
| Facilitating Condition | EFCN - LFCN | 50 | .05000 | .77756 | .10996 | .455 | .651 |
| Behavioural Intention | EHMO - LHMO | 50 | -.01000 | 1.05095 | .14863 | -.067 | .947 |
| Price Value | EPVA - LPVA | 50 | 0.13321 | 0.58648 | 0.10281 | 1.61596 | 0.11252 |
| Habit | EHAA - LHAB | 50 | -.08500 | .74848 | .10585 | -.803 | .426 |
| Self-efficacy | ESE - LSE | 50 | 0.092 | 0.86611 | 0.12249 | 0.75111 | 0.45618 |
| Behavioural Intention | EBI - LBI | 50 | .16000 | 1.12798 | .15952 | 1.003 | .321 |
| Use Behaviour | EUB - LUB | 50 | .15000 | 1.04613 | .14795 | 1.014 | .316 |
| Perceived Behavioural Control | EPBC - LPBC | 50 | -0.0856 | 0.63675 | 0.10992 | -0.9739 | 0.33487 |
| Subjective Norm | ESN - LSN | 50 | .03220 | 1.27480 | .18028 | .179 | .859 |
| Hedonic Motivation | EHMO - LHMO | 50 | -0.2172 | 0.40082 | 0.07655 | -0.9278 | 0.26834 |
| Attitude | EAT - LAT | 50 | .02700 | 1.29516 | .18316 | .147 | .883 |

**Appendix Table A2:** Common Method Bias

| Factor | Initial Eigenvalues | | | Extraction Sums of Squared Loadings | | |
| --- | --- | --- | --- | --- | --- | --- |
|  | Total | % of Variance | Cumulative % | Total | % of Variance | Cumulative % |
| 1 | 20.611 | 36.160 | 36.160 | 19.649 | 34.471 | 34.471 |
| 2 | 3.836 | 6.730 | 42.890 |  |  |  |
| 3 | 3.518 | 6.172 | 49.062 |  |  |  |
| 4 | 3.270 | 5.736 | 54.799 |  |  |  |
| 5 | 2.751 | 4.827 | 59.626 |  |  |  |
| 6 | 2.439 | 4.280 | 63.905 |  |  |  |
| 7 | 2.297 | 4.030 | 67.936 |  |  |  |
| 8 | 2.182 | 3.828 | 71.763 |  |  |  |
| 9 | 2.010 | 3.526 | 75.289 |  |  |  |
| 10 | 1.834 | 3.218 | 78.507 |  |  |  |
| 11 | 1.644 | 2.884 | 81.391 |  |  |  |
| 12 | 1.451 | 2.546 | 83.937 |  |  |  |
| 13 | 1.279 | 2.244 | 86.181 |  |  |  |
| 14 | 1.196 | 2.098 | 88.279 |  |  |  |
| 15 | .852 | 1.495 | 89.774 |  |  |  |
| 16 | .438 | .768 | 90.542 |  |  |  |
| 17 | .406 | .712 | 91.253 |  |  |  |
| 18 | .334 | .586 | 91.839 |  |  |  |
| 19 | .315 | .552 | 92.391 |  |  |  |
| 20 | .302 | .530 | 92.921 |  |  |  |
| 21 | .270 | .474 | 93.395 |  |  |  |
| 22 | .248 | .435 | 93.830 |  |  |  |
| 23 | .224 | .394 | 94.223 |  |  |  |
| 24 | .207 | .363 | 94.587 |  |  |  |
| 25 | .184 | .323 | 94.910 |  |  |  |
| 26 | .179 | .314 | 95.224 |  |  |  |
| 27 | .169 | .296 | 95.520 |  |  |  |
| 28 | .160 | .281 | 95.801 |  |  |  |
| 29 | .155 | .272 | 96.073 |  |  |  |
| 30 | .145 | .254 | 96.327 |  |  |  |
| 31 | .140 | .245 | 96.572 |  |  |  |
| 32 | .132 | .231 | 96.803 |  |  |  |
| 33 | .130 | .227 | 97.030 |  |  |  |
| 34 | .122 | .214 | 97.245 |  |  |  |
| 35 | .118 | .206 | 97.451 |  |  |  |
| 36 | .112 | .197 | 97.648 |  |  |  |
| 37 | .110 | .192 | 97.841 |  |  |  |
| 38 | .107 | .187 | 98.028 |  |  |  |
| 39 | .105 | .185 | 98.213 |  |  |  |
| 40 | .095 | .166 | 98.379 |  |  |  |
| 41 | .090 | .157 | 98.536 |  |  |  |
| 42 | .088 | .154 | 98.690 |  |  |  |
| 43 | .081 | .143 | 98.833 |  |  |  |
| 44 | .073 | .128 | 98.960 |  |  |  |
| 45 | .068 | .119 | 99.079 |  |  |  |
| 46 | .064 | .113 | 99.192 |  |  |  |
| 47 | .063 | .110 | 99.303 |  |  |  |
| 48 | .061 | .107 | 99.410 |  |  |  |
| 49 | .055 | .097 | 99.507 |  |  |  |
| 50 | .051 | .090 | 99.597 |  |  |  |
| 51 | .048 | .084 | 99.681 |  |  |  |
| 52 | .041 | .072 | 99.753 |  |  |  |
| 53 | .038 | .066 | 99.819 |  |  |  |
| 54 | .033 | .059 | 99.877 |  |  |  |
| 55 | .029 | .051 | 99.929 |  |  |  |
| 56 | .027 | .048 | 99.976 |  |  |  |
| 57 | .014 | .024 | 100.000 |  |  |  |

Extraction Method: Maximum Likelihood

**Appendix Table A3:** Full Collinearity Tests

|  | **AT** | **BI** | **EEX** | **HA** | **NCP** | **PEX** | **PV** | **SE** | **UB** |
| --- | --- | --- | --- | --- | --- | --- | --- | --- | --- |
| **AT** | -- | 1.838 | 2.197 | 2.19 | 2.204 | 2.185 | 2.184 | 2.185 | 2.179 |
| **BI** | 1.995 | -- | 1.791 | 2.208 | 1.947 | 2.305 | 1.459 | 2.488 | 1.931 |
| **EEX** | 1.208 | 1.15 | -- | 1.209 | 1.211 | 1.209 | 1.208 | 1.2 | 1.206 |
| **HA** | 2.167 | 1.881 | 2.16 | -- | 2.142 | 2.155 | 2.168 | 2.176 | 2.164 |
| **NCP** | 1.341 | 1.309 | 1.315 | 1.343 | -- | 1.339 | 1.341 | 1.333 | 1.344 |
| **PEX** | 1.929 | 1.715 | 1.946 | 1.941 | 1.943 | -- | 1.928 | 1.948 | 1.921 |
| **PV** | 2.179 | 1.971 | 2.189 | 2.185 | 2.186 | 2.165 | -- | 2.187 | 2.187 |
| **SE** | 1.631 | 1.481 | 1.626 | 1.642 | 1.646 | 1.642 | 1.642 | -- | 1.638 |
| **UB** | 1.79 | 1.487 | 1.795 | 1.801 | 1.803 | 1.778 | 1.799 | 1.795 | -- |
